# Anti-nociceptive and Anti-inflammatory Activities of Asparacosin A Involve Selective Cyclooxygenase 2 and Inflammatory Cytokines Inhibition: An *in-vitro, in-vivo*, and *in-silico* Approach

**DOI:** 10.3389/fimmu.2019.00581

**Published:** 2019-03-26

**Authors:** Nasiara Karim, Inamullah Khan, Waheed Khan, Imran Khan, Ajmal Khan, Sobia Ahsan Halim, Hizbullah Khan, Javid Hussain, Ahmed Al-Harrasi

**Affiliations:** ^1^Department of Pharmacy, University of Malakand, Chakdara, Pakistan; ^2^Department of Pharmacy, University of Peshawar, Peshawar, Pakistan; ^3^Department of Pharmacy, University of Swabi, Swabi, Pakistan; ^4^Natural and Medical Sciences Research Center, University of Nizwa, Nizwa, Oman; ^5^Department of Biological Sciences & Chemistry, College of Arts and Sciences, University of Nizwa, Nizwa, Oman

**Keywords:** Asparacosin A, acetic acid-induced writhing test, formalin test, carrageenan-induced paw edema, xylene-induced ear edema, docking studies

## Abstract

Triterpenes possess anti-inflammatory and anti-nociceptive effects. In this study anti-inflammatory activities of Asparacosin A were evaluated' using *in-vitro* cyclooxygenases 1 and 2 (COX-1/2) inhibition assays. Moreover, anti-nociceptive activities were assessed *in-vivo* by carrageenan-induced paw edema test, xylene-induced ear edema tests, and acetic acid-induced writhing and formalin tests. Additionally molecular docking was conducted to elucidate the binding mechanism of the compound and to correlate the *in-vitro* findings with the *in-silico* data. Oral administration of Asparacosin A at the doses of 10, 20, and 40 mg/kg induced significant anti-inflammatory effects (^*^p < 0.05, ^**^p < 0.01, and ^***^p < 0.001) in a dose-dependent manner in both models. Asparacosin A also inhibited the human recombinant COX-2 enzyme and caused a dose-dependent decrease in the levels of TNF-α, IL-1β, and PGE2 in the carrageenan-induced paws. Moreover, Asparacosin A displayed significant anti-nociceptive effects (^*^p < 0.05, ^**^p < 0.01, ^***^p < 0.001) at the doses of 10, 20, and 40 mg/kg in acetic-acid induced writhing test. However, in formalin test, Asparacosin A (10–40 mg/kg, p.o) produced anti-nociceptive effects only in the late phase, similar to the effect observed with the reference drug celecoxib (50 mg/kg, p.o). Molecular docking was carried out on both COX-1 and COX-2 structures which revealed that Asparacosin A targets allosteric binding site similar to the binding mode of the selective COX inhibitor. In conclusion, Asparacosin A exhibits anti-inflammatory and peripheral anti-nociceptive activities which are likely mediated *via* inhibition of COX-2 enzyme and inflammatory cytokines. Furthermore, Asparacosin A can serve as a model to obtain new and more selective potent anti-inflammatory and anti-nociceptive drugs.

## Introduction

Inflammation is an immunological defense mechanism against injury, burns, allergies, or microbial infections (1). It plays a significant role in the pathophysiology of various diseases including diabetes, neurodegenerative cardiovascular diseases, and cancer (2). Acute inflammation is a rapid process and plays an important role in the healing of wounds, whereas chronic inflammation is a sustained long-lasting pain which mainly occurs in cancer, rheumatoid arthritis, osteoarthritis and other disorders (3). Inflammation is a complex phenomenon and involves the interplay of complex reactions including activation of enzymes and release of various chemical mediators, cell migration, the release of fluids, and tissue damage and repair (4). The classical symptoms of inflammation include redness, swelling, pain, and fever. These symptoms are a result of various inflammatory mediators and chemical agents such as prostaglandins (PGs), histamine, serotonin, bradykinin, leukotrienes, and nitric oxide which are produced either locally or infiltrate in the area of insult (5). Macrophages play an important role in inflammation and are activated by various inflammatory mediators such as PGE2, tumor necrosis factor-α (TNF-α), interleukin-1β (IL-1β), interleukin-6 (IL-6), and interleukin-10 (IL-10) (6). Cyclooxygenase (COX) enzyme catalyzes the biosynthesis of PGs. There are at least two main isoforms, COX-1, and COX-2 (5). Although both isoforms catalyze the same biochemical transformation, their expression is differentially regulated. COX-1 is a constitutive enzyme and augments the physiological role of prostaglandins (PGs) including maintaining the integrity of the GIT mucosa and adequate vascular homeostasis whereas, COX-2 is induced only after an inflammatory stimulus (7, 8). Inhibition of COX enzyme is considered to be an important target in the discovery of anti-inflammatory and anti-nociceptive drugs.

Non-steroidal anti-inflammatory drugs (NSAIDs) are most widely prescribed drugs to treat inflammatory and pain disorders. This class includes drugs such as celecoxib, rofecoxib, meloxicam, diclofenac, indomethacin, and ibuprofen (9). NSAIDs exert its pharmacological activity *via* suppression of prostaglandin biosynthesis by inhibiting the enzyme cyclooxygenase (COX). It is well known that the gastrointestinal adverse effects associated with the long terms use of NSAIDs are due to inhibition of COX-1, and the adverse effects on the cardiovascular system are due to inhibition of COX-2 enzymes (10). Some COX-2 selective inhibitors including rofecoxib and valdecoxib were withdrawn from the market due to their relatively high specificity for COX-2 enzyme and subsequent cardiovascular adverse effects (11). Celecoxib is the only coxib currently approved by US food and drug administration (FDA), possess less COX-2 selectivity than other drugs in the same class and has some degree of COX-1 inhibitory effects. Thus, the focus is on the development of anti-inflammatory drugs with greater selectivity for COX-2 enzymes with some degree of COX-1 inhibition.

**Figure 1:**
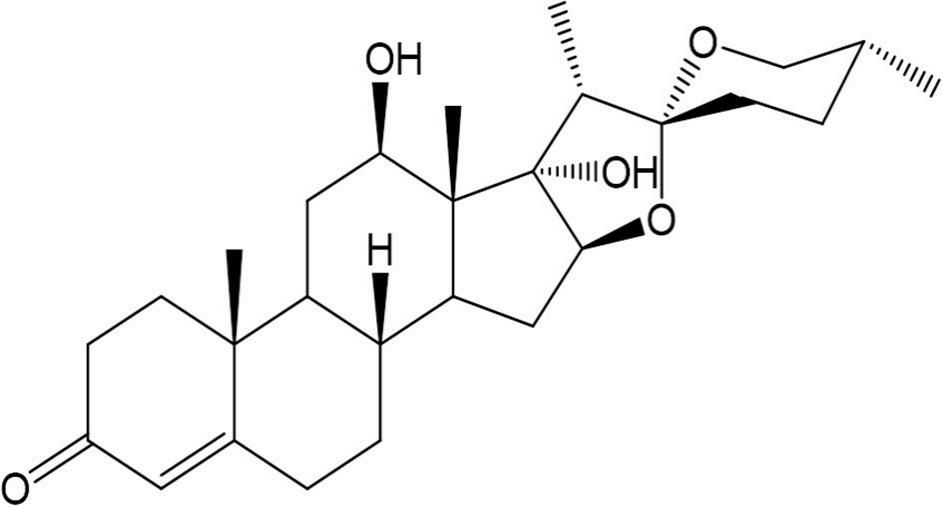
Chemical structure of Asparacosin A.

Anti-inflammatory agents from plant sources have been extensively studied, and have shown promising results against inflammatory disorders due to their mild adverse effects (12–14). In this context, a triterpenoid compound, Asparacosin A (Figure 1) previously isolated from *Asparagus racemosus* (15), was selected based on pilot scale studies to explore its anti-inflammatory activity in *in-vitro* cyclooxygenase 1 and 2 (-1/2) assays followed by *in-vivo* studies using animal models of inflammation. Additionally, the effects of Asparacosin A on IL-1B, TNF-α, and PGE2 levels were also determined following carrageenan-induced paw edema. Furthermore, the anti-nociceptive effects of Asparacosin A was determined using mouse models of pain. *In-silico* docking experiment was carried out to correlate the binding affinities with these *in-vitro* anti-inflammatory activities.

## Materials and Methods

### Chemicals and Drugs

Normal saline (Searle Pharma), celecoxib, carrageenan, formalin, gum acacia, and acetic acid were purchased from Sigma (St. Louis, MO, USA). All the compounds and chemicals were of analytical grade. Asparacosin A was isolated and purified as reported earlier (15). Asparacosin A was subjected to stability testing in PBS using High performance liquid chromatography (HPLC) (Figure S1). IL-1β, TNF-α, and PGE2 kits were purchased from Shanghai Yanjin Biological Technology Co., Ltd. (Shanghai, China).

### Animals

Swiss mice in the weight range of 20–30 g and Sprague Dawley rats in the weight range of 150–220 g were purchased from the National Institution of Health, Islamabad, Pakistan. Animals were kept in the Department of Pharmacy, University of Malakand's animal house having access to fresh water and standard food *ad libitum*. The animals were maintained at 12 h light and dark cycle and with room temperature maintained at 22–25°C in the animal house. All procedures involving animals have been approved by the Animal Ethical Committee of the Department of Pharmacy, University of Malakand (DAEC/PHARM/2016/11) and were conducted according to the guidelines of the UK animal scientific procedure act, 1986.

### Acute Toxicity

The acute toxicity of Asparacosin A (1–200 mg/kg) was determined by using mice according to the previously described method (16). The animals were divided into five groups (*n* = 6). Group I served as a control and received normal saline with 2% gum acacia suspension (vehicle) orally. Groups II, III, IV and V have intraperitoneally (i.p.) received Asparacosin A 50, 100, 150, and 200 mg/kg of Asparacosin A, respectively. Each animal was subjected to various parameters including writhings, changes in respiration, hypersensitivity, convulsions, lacrimation, salivation, ataxia, body temperature, spontaneous activity, and catalepsy 30 min prior to injection (baseline) and monitored for next 3-days after drug administration for any kind of behavioral, physical, and pharmacological toxic effects.

## Anti-inflammatory Activity

### Carrageenan-Induced Edema

Carrageenan-induced hind paw edema is the most commonly used animal model to evaluate the anti-inflammatory potential of pharmacological substances (17). In this test, rats were divided into six groups with each group containing 8 animals. The control group was given vehicle (10 ml/kg p.o). Groups II-V were given Asparacosin A at the doses of 1, 10, 20, and 40 mg/kg (p.o) whereas the animals in group VI received celecoxib (50 mg/kg, p.o). After 1 h, acute inflammation was induced in the right hind paw of rats by subplantar injection of 0.1 ml carrageenan (1% suspension of carrageenan (0.1 mL) using 2% gum acacia as a suspending agent in normal saline). The paw volume was measured at 0, 3, and 5 h plethysmometrically after the carrageenan injection. The percentage inhibition of the inflammation was determined by the formula; %*I* = 1–(d*t*/d*c*) × 100, where “d*t*” is the difference in paw volume in the drug-treated group and “d*c*” is the difference in paw volume in control group. Furthermore, “*I*” stands for inhibition of inflammation.

### Determination of the IL-1β, TNF-α, and PGE2 Levels in Rat Paw

The level of inflammatory mediators including IL-1β, TNF-α, and PGE2 in rat paw tissues was determined according to the method described previously (18). Five hours after carrageenan administration, rats were sacrificed and the paw tissues were collected and weighed. The tissues were subsequently snap frozen in liquid nitrogen and stored at −80°C. On the day of the experiment, paw tissues were homogenized in phosphate buffer (10% w/v, 0.1 M PBS, PH = 7.4). The homogenates were centrifuged at 18,000 rpm for 25 min at 4°C. The supernatants were collected and the levels of IL-1β, TNF-α, and PGE2 were determined by reference to standard curves (0–1,000 ng/ml) constructed in each assay according to the protocols of respective commercial ELISA kits.

### Xylene-Induced Ear Edema

The xylene-induced ear edema was conducted according to the modified method previously described (19). Animals were segregated into six groups with eight (*n* = 8) animals per group. Group I was given vehicle (10 ml/kg p.o) which served as control. Group II, III, IV, and V were given Asparacosin A at the doses of 1, 10, 20, and 40 mg/kg (p.o), respectively. Group VI received reference drug, celecoxib 50 mg/kg (p.o) and served as positive control. After 1 h of administration, animals were given 20 μl xylene on the inner and outer surfaces of the right ear. After 1 h of xylene application, the animals were sacrificed by cervical dislocation and ear biopsies (10 mm in diameter) were cut. The degree of swelling was calculated by measuring the difference in weight of the right and left ear of the same animal and the % inhibition of swelling was calculated.

### Cyclooxygenase (COX-1 and COX-2) Inhibition Assay

The inhibitory effects of Asparacosin A on COX-1 (ovine) and COX- 2 (human recombinant) was studied by commercial colorimetric COX inhibitor screening assay kit (Cayman test kit-560131; Cayman Chemical Company) (20) Briefly, 150 μl of assay buffer, 10 μl of heme, 10 μl of COX-1 or COX-2 enzyme, and 20 μl of Asparacosin A were added into 96-well plate. The plate was shaken for 20 s on a shaker followed by incubation at 25°C for 5 min. Twenty micro liters of colorimetric substrate solution and arachidonic acid were added to start the reaction. After careful shaking, it was incubated at 25°C for 10 min. The absorbance of each well was measured at 590 nm with a microplate reader.

## Antinociceptive Activity

### Acetic Acid-Induced Pain Model

The peripheral antinociceptive activity of Asparacosin A was determined by acetic acid-induced abdominal constriction assay in mice (21). Briefly, the animals were divided into six groups, each group of six animals (*n* = 8). Group I served as control and received vehicle (10 ml/kg, p/o). Groups II, III, IV, and V groups received Asparacosin A (1, 10, 20, and 40 mg/kg) orally, respectively, whereas group VI received celecoxib 50 mg/kg (p.o). The writhes were induced by the intraperitoneal injection of 1% acetic acid, 30 min after the treatment of all groups as stated above. The numbers of writhes (muscular contractions) were counted over a period of 20 min after acetic acid injection. The number of writhes in each treated group was compared with the control (saline-treated group) and the percent inhibition of the writhes was calculated.

### Formalin Test

Formalin test was conducted according to the method described by Wang et al. (19). Mice were divided into six groups with six animals in each group. Group 1 received the vehicle and served as a control group. Groups II-V received Asparacosin A at the dose of 1, 10, 20, and 40 mg/kg (p.o), respectively. Animals in group VI received celecoxib (50 mg/kg, p.o) and served as positive control. After 35 min, each animal was administered with 20 μl of 2% formalin (in 0.9% saline, sub-plantar) into the right hind paw. The total time spent licking the injected paw was calculated and expressed as the total licking time in the early phase (0–5 min) and late phase (10–30 min) after the formalin injection.

### Molecular Docking Studies

Molecular docking was carried out by MOE docking suit (22). The three-dimensional structures of ovine COX-1 (PDB code 3KK6) and mouse COX-2 (PDB code 3LN1) were selected. For docking protein files were prepared with protonate3D utility of MOE with AMBER12: EHT force field (which has Iron parameters). The protein 3LN1 is a homotetramer, while 3KK6 is a homodimer. The chain A of both proteins was selected and water molecules that interact at the active and allosteric binding sites were retained in the protein structures, hydrogen atoms were added and partial charges were calculated. The 3D structure of Asparacosin A was prepared by Chem Draw. Hydrogen and charges were added on ligand by MOE, and the structure was minimized with AMBER12: EHT force field until the gradient was reached at 0.1 kcal/mol/A^2^. Induce fit docking protocol was applied with Alpha Triangle placement method and London dG scoring function. For pose refinement, force field refinement method and GBVI/WSA dG rescoring were used. The maximum poses of each compound were set to 30. Moreover, docking interactions were rendered by Chimera (23).

### Statistical Analysis

The data obtained were analyzed using GraphPad Prism (version 5) and were expressed as mean ± SEM. The significance of the difference between means was determined by one way ANOVA followed by Dunnett's *post hoc* test and the *p* < 0.05 values were considered significant.

## Results

### Acute Toxicity Studies

Acute toxicity studies revealed that the administration of Asparacosin A (50–200 mg/kg) did not produce any untoward changes in the general behavior of animals. Asparacosin A did not produce any convulsions, writhing, respiratory distress, changes to reflex activity or mortality. All animals seemed well with no observable changes in behavior or appearance during the 3 days of acute toxicity trials.

### Anti-inflammatory Activity of Asparacosin A in Carrageenan-Induced Paw Edema

In carrageenan-induced paw edema test, intraperitoneal injection of carrageenan in rats showed a time-dependent increase in paw edema. The maximum increase in edema was observed at the 5th hour of carrageenan administration in control group. Asparacosin A at the doses of 20 and 40 mg/kg caused significant inhibition (^**^p < 0.01) in paw edema after 3rd and 5th hour of carrageenan administration whereas 10 mg/kg of Asparacosin A reduced paw edema significantly (*p* < 0.05) after 5 h of carrageenan administration. Asparacosin A was ineffective at the dose of 1 mg/kg and did not decrease paw edema (*p* > 0.05). The standard anti-inflammatory drug celecoxib (50 mg/kg) also significantly decreased paw edema (^***^p < 0.001) after 3rd and 5th hour of carrageenan administration (Table 1).

**Table 1:** Effect of Asparacosin A on carrageenan-induced paw edema in rats.

**Treatment**	**Dose (mg/kg)**	**Increase in paw edema (ml) and % inhibition (%I)**	
		**After 3h**	**After 5h**
Control	-	0.91 ± 0.098	0.83 ± 0.061
Celecoxib	50	0.19 ± 0.021^***^ (79.1)	0.26 ± 0.018^***^ (68.7)
Asparacosin A	1	0.85 ± 0.045 (6.6)	0.82± 0.055 (1.2)
Asparacosin A	10	0.76 ± 0.054 (16.5)	0.65 ± 0.045^*^ (21.7)
Asparacosin A	20	0.45 ± 0.036^**^ (50.5)	0.47 ± 0.021^**^ (43.4)
Asparacosin A	40	0.40 ± 0.045^**^ (56.0)	0.35 ± 0.034^**^ (57.8)

*Values are expressed as mean ± SEM*.

* *p < 0.05*,

** p < 0.01, and

*** *p < 0.001, compared to the vehicle group. Difference between groups were analyzed by analysis of variance (one-way ANOVA) followed by Dunnett's test*.

### Effect of Asparacosin A on IL-1β, TNF-α, and PGE2 in Carrageenan-Induced Rat Paw Edema

**Figure 2:**
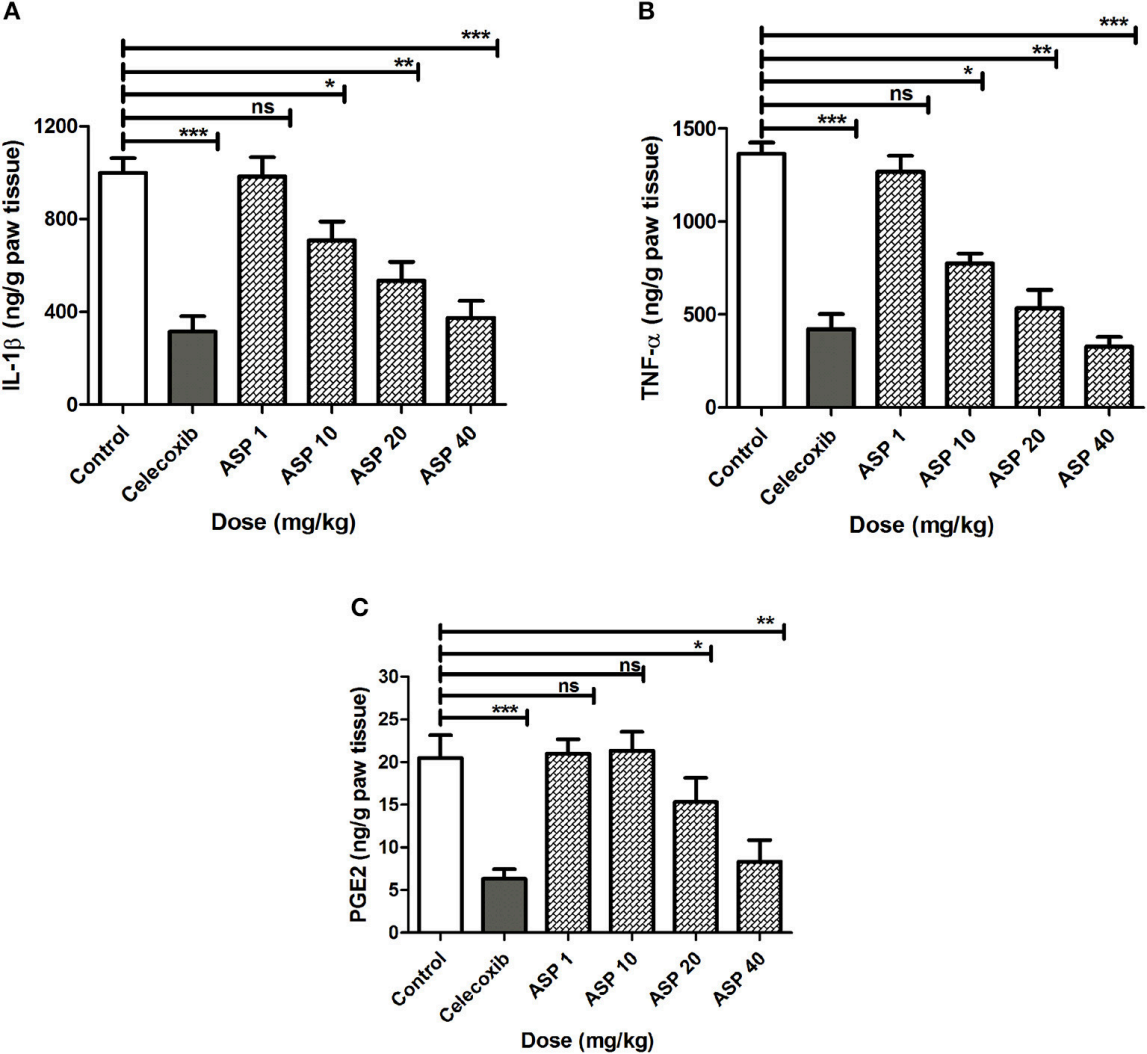
Effect of Asparacosin A on IL-1β **(A)**, TNF-α **(B)**, and PGE2 **(C)** in carrageenan-induced edema in rat hind paws. Values represent mean +SEM for each group (*n* = 8). ^*^P < 0.05, ^**^P < 0.01, ^***^P < 0.001, compared to the vehicle control group. Difference between groups were analyzed by analysis of variance (one-way ANOVA) followed by Dunnett's test.

The effects of Asparacosin A on the level of inflammatory mediators including IL-1β, TNF-α, and PGE2 in carrageenan-induced edema in rats paw is shown in Figure 2. Asparacosin A caused a significant reduction in the level of IL-1β and TNF-α compared to the vehicle control group at doses of 10, 20, and 40 mg/kg (^*^p < 0.05, ^**^ *p* < 0.01, ^***^p < 0.001). However, Asparacosin A did not significantly decreased the levels of IL-1β and TNF-α at 1 mg/kg (*p* > 0.05) (Figures 2A,B), while it significantly decreased PGE2 at doses of 20 and 40 mg/kg, respectively (^*^p < 0.05, ^**^p < 0.01) (Figure 2C). Similar results were obtained with the reference drug celecoxib which significantly decreased IL-1β, TNF-α, and PGE2 at the dose of 50 mg/kg (^***^p < 0.001).

### Anti-inflammatory Activity of Asparacosin A in Xylene-Induced Ear Edema in Rats

iThe anti-inflammatory effect of various treatments of Asparacosin A in xylene-induced ear edema is depicted in Table 2. Asparacosin A significantly inhibited edema formation induced by xylene at the doses of 10, 20, and 40 mg/kg (^*^p < 0.05, ^**^p < 0.01, ^***^p < 0.001, compared to the vehicle control group). Moreover, Asparacosin A dose-dependently decreased xylene-induced edema by 40.58, 59.08, and 71.03% at doses of 10, 20, and 40 mg/kg, respectively. Celecoxib at the dose of 50 mg/kg also caused a significant reduction (74.04%) in edema formation compared to control (^***^p < 0.001). The anti-inflammatory effect of Asparacosin A at the highest dose tested (40 mg/kg) was comparable to the effect of celecoxib (50 mg/kg).

**Table 2:** Effect of Asparacosin A on xylene induced- ear edema in rats.

**Treatment**	**Dose (mg/kg)**	**Swelling (%)**	**Inhibition (%)**
Vehicle	-	60.25 ±4	-
Celecoxib	50	15.64 ± 5	74.04^***^
Asparacosin A	1	51.62 ± 6	14.32
Asparacosin A	10	35.8 ± 4	40.58^**^
Asparacosin A	20	24.65 ± 6	59.08^**^
Asparacosin A	40	17.45 ± 3	71.03^***^

*Values are expressed as mean ± SEM*.

* *p < 0.05*,

** p < 0.01, and

*** *p < 0.001, compared to the vehicle group. Difference between groups were analyzed by analysis of variance (one-way ANOVA) followed by Dunnett's test*.

### *In vitro* Cyclooxygenase Inhibition Assay

**Figure 3:**
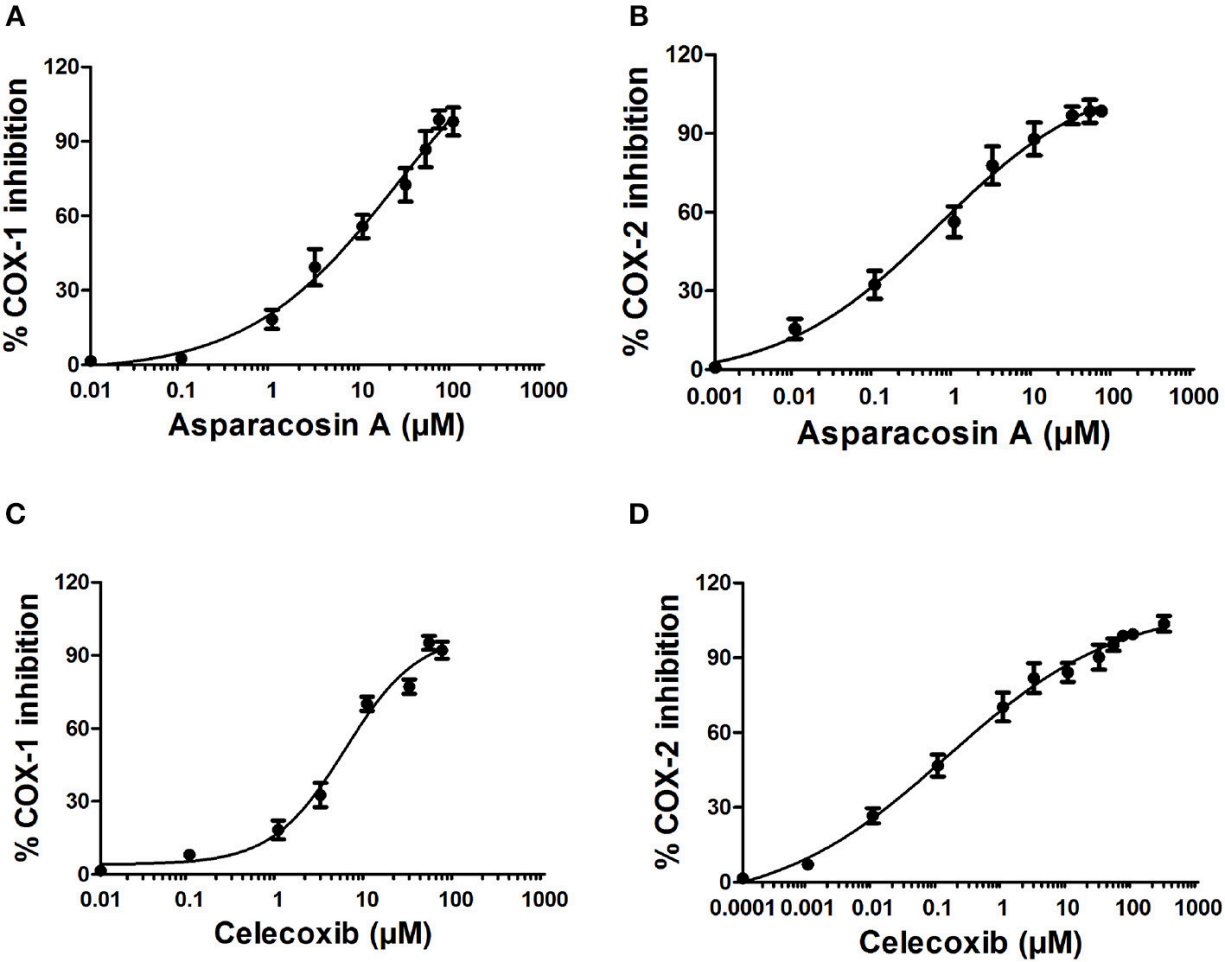
Effects of Asparacosin A on COX-1 **(A)**, and COX-2 **(B)** and celecoxib on COX-1 **(C)**, and COX-2 **(D)** enzymes. The IC_50_ were determined using nonlinear fit and sigmoidal concentration-inhibition curves. The concentration used for the calculation of IC_50_ values ranged from 0.0001 to 300 μM.

The inhibitory potential of Asparacosin A against COX-1/2 enzymes was evaluated by enzyme immune assay (EIA) method against ovine COX-1 and human recombinant COX-2. Asparacosin A inhibited COX-1 enzyme with IC_50_ value of 24.2 μM (Figure 3). In contrast, the IC_50_ value against COX-2 was 0.6 μM with a selectivity index of 40.3 μM (Table 3, Figure 3). Similarly, the standard reference drug celecoxib inhibited COX-1 and COX-2 enzymes with IC_50_ values of 5.6 and 0.1 μM, respectively with a selectivity index of 56 (Table 3, Figure 3). These data for the standard drug are consistent with the literature reports.

**Table 3:** Experimentally determined IC_50_ values of Asparacosin A against COX-1 and COX-2 enzymes.

**Treatments**	**IC_**50**_ (μM) COX-1**	**IC_**50**_ (μM) COX-2**	**Selectivity index (SI) IC_**50**_ (COX1)/ IC_**50**_ (COX2)**
Asparacosin A	24.2	0.6	40.3
Celecoxib	5.6	0.1	56

### Assessment of Antinociceptive Effects of Asparacosin A in Acetic Acid-Induced Writhing Test

**Figure 4:**
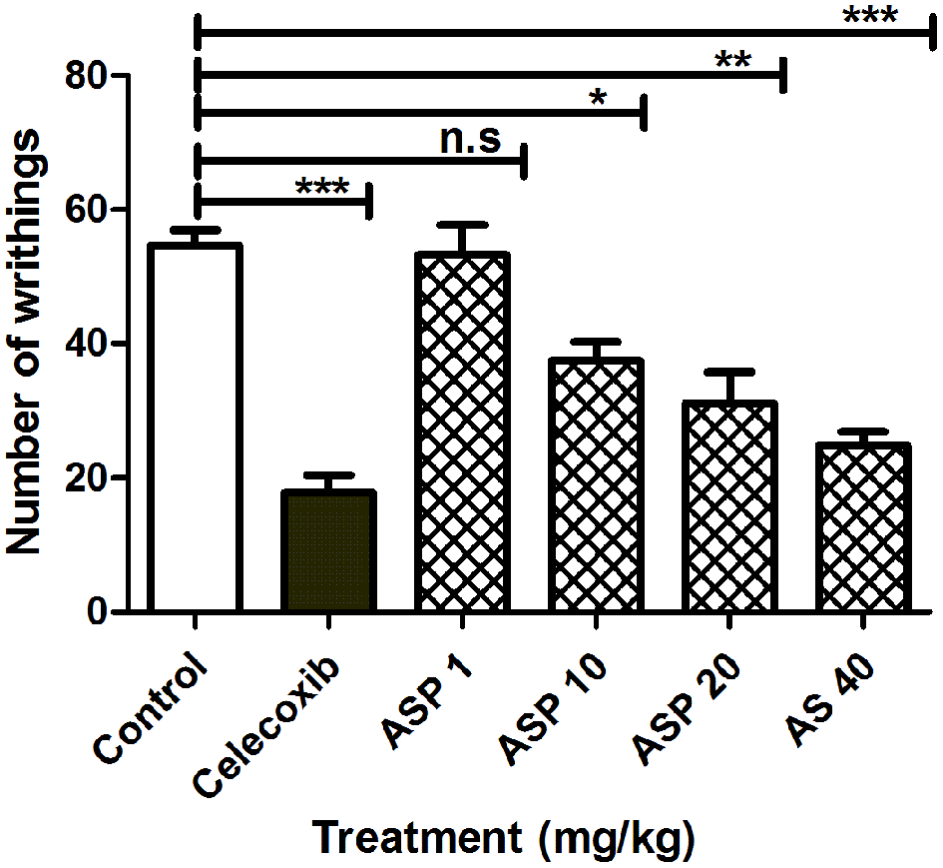
Effect of Asparacosin A (1–40 mg/kg, p.o) on number of writhings in acetic induced writhing test in mice. Values represent mean + SEM for each group (*n* = 8). ^*^P < 0.05, ^**^P < 0.01, ^***^P < 0.001, compared to the vehicle control group.

When 1%(v/v) acetic acid solution (3.3 ml/kg body weight) was injected intraperitoneally in mice, the control animal showed 56.26 ± 4.76 writhing count. Oral administration of celecoxib (50 mg/kg) caused a significant reduction in the number of writhes (^***^p < 0.001; ANOVA with Dunnett's *post hoc* test, *n* = 8). The writhing count was decreased from 56.26 ± 4.76 to 17.82 ± 2.43. Asparacosin A caused significant reduction of the writhing count compared to vehicle control at the doses of 10, 20, and 40 mg/kg (^*^p < 0.05, ^**^p < 0.01, ^***^p < 0.001; *n* = 8). The inhibition was dose-dependent and the highest reduction in the writhing count was observed at 40 mg/kg (Figure 4). Asparacosin A was found to be ineffective at the dose of 1 mg/kg (*p* > 0.05). The reduction in the writhing count observed with Asparacosin A at the dose of 40 mg/kg was similar to the reduction in writhing observed with celecoxib at 50 mg/kg, indicating similar potency and efficacy.

### Assessment of Antinociceptive Effects of Asparacosin A in Formalin Test

**Figure 5:**
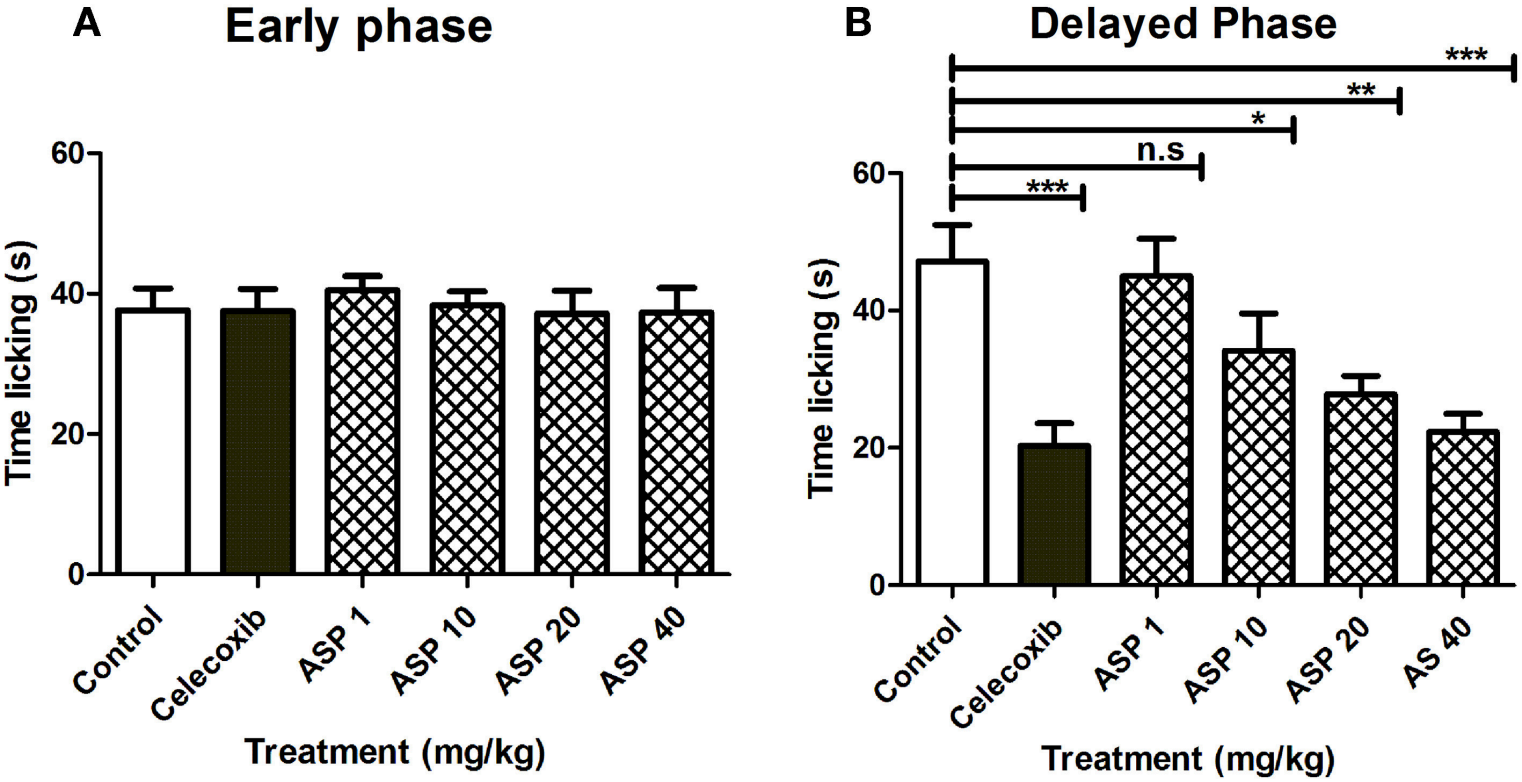
Effect of Asparacosin A (1–40 mg/kg, p.o) on paw licking in early phase **(A)**, and delayed phase **(B)** after intraplantar injection of formalin in formalin injection test. Values represent mean + SEM for each group (*n* = 8). ^*^P < 0.05, ^**^P < 0.01, ^***^P < 0.001, compared to the vehicle control group.

Figure 5 shows the effects of treatment of vehicle, celecoxib or Asparacosin A in formalin-induced nociceptive response. The results showed that Asparacosin A at the doses of 10–40 mg/kg caused a significant reduction in paw licking time (^*^p < 0.05, ^**^p < 0.01, ^***^p < 0.001; ANOVA with Dunnett's *post hoc* test, *n* = 8) in the delayed phase of formalin test. In contrast, Asparacosin A (1–40 mg/kg) did not cause a significant decrease in time, the mice took licking the injected paw (*p* > 0.05) in the early phase. Similar results were obtained with celecoxib (50 mg/kg, p.o) which also significantly decreased time taken by mice licking the injected paw (^***^p < 0.001; *n* = 8) only in the delayed phase of formalin test. No significant effect was observed on nociception with celecoxib in the early phase (*p* > 0.05).

### Docking Simulations

**Figure 6:**
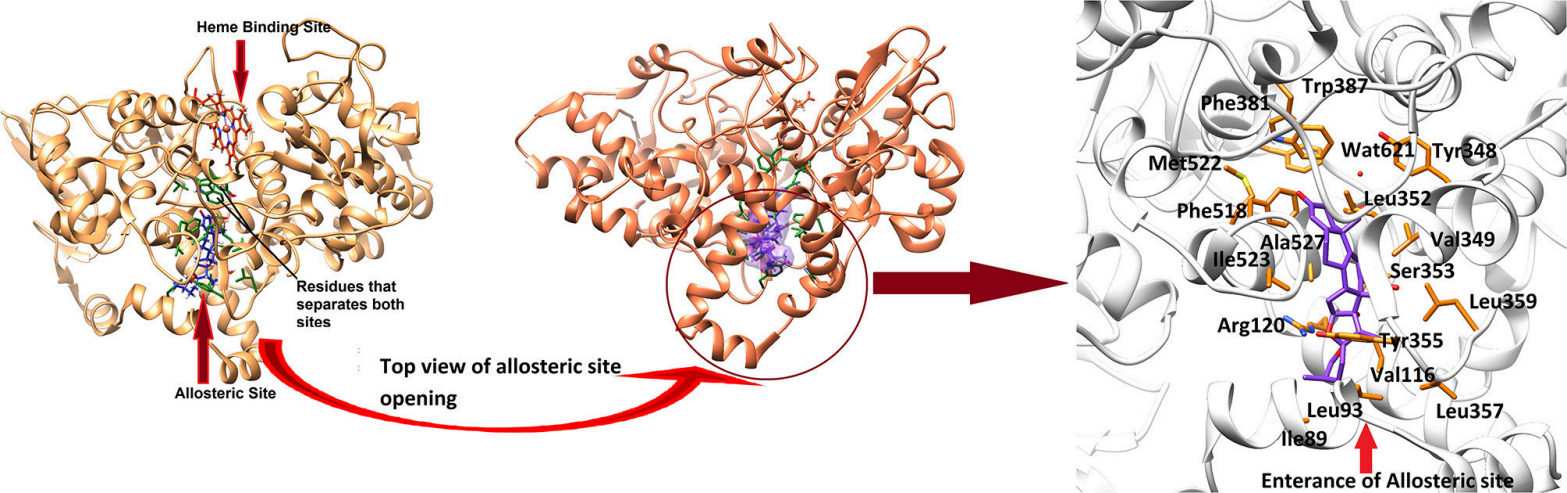
The 3D-structure of ovine COX-1 (PDB code: 3KK6). The heme (orange sticks) binding site and the allosteric sites (AS) are presented. Ligand (purple sticks) binds at AS. The top view of the AS shows that the entrance loop has a wide opening which accommodates the ligand in AS. The AS residues are shown in green sticks. The binding mode of Asparacosin A in the AS of ovine COX-1 is depicted. The surrounding residues are shown in orange sticks.

**Figure 7:**
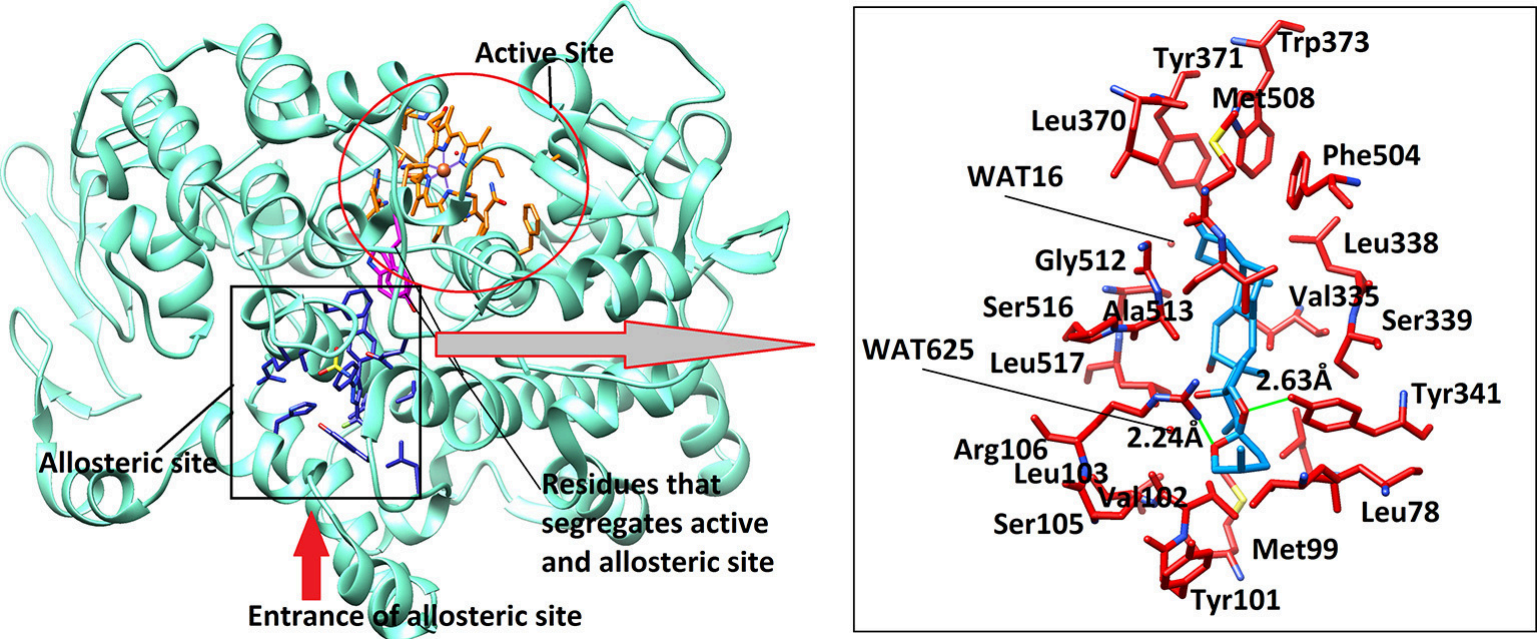
The 3D-structure of mouse COX-2 (PDB code: 3LN1). The heme (orange sticks) binding site and the allosteric site (AS) is presented. Ligand (blue sticks) binds at AS. Residues which separates two binding sites are shown in magenta sticks. The binding mode of Asparacosin A in the AS of mouse COX-2 is highlighted in box, where surrounding residues are shown in orange-red sticks. Hydrogen bonds are shown in green lines and their distances are labeled. Water molecules do not play role in protein-ligand bridging.

In order to study the mechanism of COX inhibition of Asparacosin A, molecular docking strategy was applied. The compound was docked into the binding sites of ovine COX-1 and mouse COX-2. The cyclooxygenase enzyme has two binding sites: an active site and an allosteric site. The active site of ovine COX-1 contains an iron-containing heme molecule, surrounded by 17 residues. The surrounding residues are tabulated in Table 4. The heme molecule forms a hydrogen bond with Thr212 (2.72 Å) in the active site of ovine COX-1. The allosteric binding site is segregated from active site by a closed conformation of Phe381, Tyr385, and Trp387. The conformations of Phe381, Tyr385, and Trp387 is tilted toward heme molecule, thus do not allow heme moiety to escape and the allosteric site is spatially separated. The distance between Heme moiety and the allosteric binding site is ~10–13 Å. The allosteric site residues are tabulated in Table 4. Celecoxib is an inhibitor of both COX-1 and COX-2 which binds at the allosteric site. The allosteric site of ovine COX-1 is constituted by 19 residues among which Gln192 and Leu352 forms hydrogen bonds with celecoxib at a distance of 2.33 and 2.24 Å, respectively. The rest of the residues provide hydrophobic interactions to celecoxib. The heme binding site and the allosteric site is depicted in Figure 6. The opening of the allosteric site shows that it is wide enough to accommodate this ligand. The binding mode of Asparacosin A showed that it binds at the allosteric binding site, thus depicting the allosteric modulation of the protein. However, molecule formed bidentate interactions with the side chain of Arg120 which do not form a hydrogen bond with celecoxib. The H-bond distance between the O1, and O7 of Asparacosin A and NE and NH2 of Arg120 is 2.74 and 1.87 Å, respectively. Thus, Arg120 acted as H-bond donor to the compound. Moreover, the side chains of Ile89, Leu93, Val116, Tyr348, Val349, Leu352, Ser353, Tyr355, Leu357, Ley359, Phe381, Trp387, Phe518, Met522, Ile523, Gly526, Ala527, Ser530, and Leu531 provides hydrophobic interactions to the compound, and the hydrophobic nature of allosteric site forms a cage to grab the ligand molecule inside the allosteric binding site of COX-1, where Leu89, Leu93, Val116, and Tyr355 are located at the entrance of the allosteric site, thus molecule is fixed in the allosteric site via hydrophobic interactions. The binding mode of Asparacosin A is depicted in Figure 7. We also observed that water molecules are not involved in protein-ligand bridging, therefore it may presume that there is no role of water molecules in the allosteric site.

**Table 4:** The active site and allosteric site residues of ovine COX-1 and mouse COX-2 proteins.

**Active site in ovine COX-1**	**Active site in mouse COX-2**	**Allosteric site in ovine COX-1**	**Allosteric site in mouse COX-2**
TYR148	TYR134	VAL349	VAL335
ALA199	ALA185	**LEU352**	**LEU338**
PHE200	PHE186	SER353	**SER339**
ALA202	ALA188	TYR355	TYR341
GLN203	GLN189	LEU359	LEU345
HIS207	HIS193	TYR385	TYR371
PHE210	PHE196	TRP387	TRP373
LYS211	LYS197	SER516	ALA502
**THR212**	**THR198**	ILE517	ILE503
LEU295	VAL281	PHE518	PHE504
—	**HIS200**	MET522	MET508
ASN382	**ASN368**	ILE523	VAL509
TYR385	TYR371	GLY526	GLY512
HIS386	HIS372	ALA527	ALA513
TRP387	TRP373	LEU351	LEU517
HIS388	**HIS374**	HIS90	HIS75
MET391	LEU377	ARG120	ARG106
HIS446	—	—	**ARG499**
VAL447	VAL433	VAL116	VAL102
ASP450	—	**GLN192**	GLN178
LEU408	LEU394	SER530	SER516

*The conserved residues (depicted with their sequence number) are highlighted. The residues involved in H-bonding with the Heme and Celecoxib are shown in Bold*.

The active site of mouse COX-2 also contains iron bonded heme molecule, surrounded by ~18 residues (Table 4), among them three residues forms hydrogen bonds with the heme moiety. Thr198, His200, and Asn368 mediate three, two and one hydrogen bond with heme, respectively, while other residues provide hydrophobic interactions to the molecule. Two of the active site residues (Tyr371 and Trp373) segregate the active site from the allosteric site, however in mouse COX-2 structure the conformation of these two residues is titled more toward the allosteric site. This indicates that these residues might have gatekeeping role between active and allosteric sites. The allosteric site of mouse COX-2 structure (PDB: 3LN1) is complexed with celecoxib, which mediates four hydrogen bonds with Gln178, Leu338, Ser339, and Arg499 with the bond length of 3.08, 2.71, 2.96, and 3.34 Å, respectively. The docking results showed that Asparacosin A does not mediate hydrogen bonding with any of these residues. The binding mode of Asparacosin A revealed that the compound was bound at the entrance of the allosteric site and formed two hydrogen bonds with the side chains of Arg106 and Tyr341. The NH1 of guanidinium moiety of Arg106 formed H-bond with the sugar oxygen of the compound at a distance of 2.24 Å, while the side chain –OH of Tyr341 mediated H-bond with the furane oxygen of the compound (2.63 Å). The residues Leu345, Val335, Val509, and Leu517 provides strong hydrophobic interactions to the molecule. Additionally, Tyr371, Trp373, and Phe504 stabilize the compound in the allosteric site by π-π interactions. The docked view of the compound is depicted in Figure 7. We again observed that there is no role of water molecules in protein-ligand bridging in the allosteric site of mouse COX-2 enzyme.

The docking results are in agreement with the experimental observations. The compound most dominantly has specificity toward mouse COX-2 enzyme than the ovine COX-1 enzyme. The docking energies confirms our experimental findings (Table 5). The compound binds with COX-2 enzyme with ~4-folds higher binding energies than COX-1 enzyme. Moreover, the docking results also depict that the compound has a lower potential than the standard compound celecoxib. The reported inhibitor celecoxib fits deeply into the allosteric site, while compound Asparacosin A binds at the entrance of the allosteric site, blocking its function. This could be the reason of higher energies of celecoxib compared to Asparacosin A. We observed that Arg120 and Arg106 played important role in ligand binding in the allosteric site of ovine COX-1 and mouse COX-2 enzymes, respectively.

**Table 5:** Docking results of Asparacosin A and Celecoxib in Ovine COX-1 and Mouse COX-2 enzymes.

**Compounds name**	**Docking scores**	
	***Ovine* COX-1**	***Mouse* COX-2**
Asparacosin A	−2.53	−9.29
Celecoxib (standard)	−8.96	−11.87

## Discussion

The development of new, effective, and safer alternative anti-inflammatory and anti-nociceptive agents to NSAIDS is a major challenge. The anti-inflammatory and anti-nociceptive drugs with high selectivity for COX-2 enzyme with no or less affinity for COX-1 enzymes are considered to be safer as they are devoid of unwanted gastrointestinal side effects associated with the housekeeping enzyme COX-1 inhibition (2). Natural products from medicinal plants have been considered as an alternative potential source of pharmacological substances with minimal adverse effects (4).

In this study, Asparacosin A was found to possess preferential selectivity toward inhibiting COX-2 enzyme over COX-1. The IC_50_ value of Asparacosin A against COX-2 was 50-fold less compared to COX-1 inhibition with a selectivity index of 40.3. Under similar conditions, the reference drug celecoxib also showed significant inhibitory effects against COX-2 enzyme compared to COX-1 with a selectivity index of 56. These data for the reference drug is consistent with the literature (20, 24).

Based on the results of *in-vitro* enzyme inhibition assays, Asparacosin A was evaluated for it's *in -vivo* anti-inflammatory effects using carrageenan-induced paw edema in rats. The carrageenan-induced rat paw edema assay has frequently been used to evaluate the anti-inflammatory effect of natural products. The development of carrageenan-induced paw edema is a biphasic phenomenon in nature (25). The first phase (1–2 h after carrageenan injection) is mediated by histamine, serotonin, and bradykinins which are released from mast cells into the surrounding damaged tissues. The second phase (3–6 h after carrageenan injection) of inflammatory reaction is associated with the release of arachidonate metabolites such as prostaglandins, leukotrienes, and various cytokines such as IL-1β, IL-6, IL-10, and TNF-α (25, 26). In this study, the oral administration of Asparacosin A at the doses of 20 and 40 mg/kg displayed significant inhibition of paw edema in rats 3 h after carrageenan injection and this effect continued for up to 5 h. Asparacosin A (at dose 10 mg/kg) significantly inhibited paw edema only after 5 h. The inflammatory mediators such as PGE2, and various cytokines including IL-1β, IL-6, and TNF-α are fundamental players of the inflammatory response (27–29). Mononuclear phagocytes produce TNF-α which in turn cause an immune response by stimulating T cells and macrophages and cause the secretion of other inflammatory cytokines (18). Activated NF-κB, an important transcription factor, causes up-regulation of the expression of proinflammatory cytokine genes including TNF-α, IL-1β, and IL-6 (27). Furthermore, IL-1β and TNF-α induce cyclooxygenase 2 (COX-2). COX-2 catalyzes the synthesis of PGE2. Our results showed that Asparacosin A inhibited the production of TNF-α, IL-1β, and IL-6 significantly, inhibited COX-2 with preferential selectivity and decreased carrageenan-induced paw edema in rats. These results suggest that Asparacosin A act in the second phase of edema development.

Acetic acid-induced writhing and formalin tests were used to evaluate the *in vivo* antinociceptive effects of Asparacosin A. Acetic acid-induced writhing test is widely used model to evaluate peripheral antinociceptive effects (20). In this model, pain is generated by PGE_2_ and PGE_2_ alpha, histamine, serotonin, bradykinin, and cytokines (TNF-α, IL-1β, IL-6, and IL-8). These inflammatory mediators also increase vascular capillary permeability, decrease pain threshold, and increase the sensitivity of nerve terminals of nociceptive fibers (30–32). In this study, Asparacosin A inhibited PGE2, TNF-α, and IL-1β and exerted significant anti-nociceptive effects in acetic acid-induced writhing test in mice suggesting the involvement of these mediators in the anti-nociceptive activity of Asparacosin A.

Two distinct phases are involved in formalin test: the early phase (0–5 min) characterized by neurogenic pain which is caused by direct activation of nociceptors and the late phase (10–45) characterized by inflammatory pain, caused by inflammation and associated with inflammatory cytokines in the periphery and spinal cord sensitization (33, 34). Studies have shown that centrally acting anti-nociceptive drugs inhibit both phases of formalin-induced nociception whereas peripherally acting drugs inhibit only the late phase (33, 35). In this study, Asparacosin A alleviated only the inflammatory pain, similar to celecoxib suggesting its peripheral action only. This study showed that Asparacosin A decreased the production of PGE2 and the levels of inflammatory cytokines TNF-α, IL-1β, and IL-6 in rat paw tissue (Figure 2). The observed reduction in inflammatory pain by Asparacosin A may be due to its ability to inhibit these inflammatory mediators.

Finally, *in-silico* prediction displayed that Asparacosin A has better binding with the mouse COX-2 when compared to ovine COX-1 enzyme due to the presence of two hydrogen bonds in the mouse COX-2 structure. It may be concluded that there are certain structural features of Asparacosin A that support its role in *in-vivo* activities. The docking energies of celecoxib are higher than the new modulator in both COX-1 and COX-2 enzymes. The docking analysis was based upon the hydrogen bond and hydrophobic interactions, which demonstrated an excellent correlation with the experimental findings.

## Conclusions

In conclusion, the present study demonstrated the effectiveness of Asparacosin A in different anti-inflammatory and anti-nociceptive tests. The anti-inflammatory and anti-nociceptive effects of Asparacosin A are likely mediated by inhibiting the formation of PGs and TNF-α, IL-1β, IL-6, and−2. Thus, Asparacosin A with its potent anti-inflammatory and anti-nociceptive effects can be suggested as an effective anti-inflammatory compound. However, detailed mechanistic studies are required to substantiate the current findings.

## Author Contributions

NK, WK, and InK performed the *in-vitro* and *in-vivo* experiments. ImK performed the toxicity studies. SH performed molecular docking and computational analysis. HK and JH performed the drug stability in BPS buffer using HPLC. NK, AA-H, and AK analyzed the data and wrote the manuscript.

### Conflict of Interest Statement

The authors declare that the research was conducted in the absence of any commercial or financial relationships that could be construed as a potential conflict of interest.

## Abbreviations

COX: Cyclooxygenase
ASP: Asparacosin
TNF-α: Tumor necrosis factor- α
IL-1β: interleukin-I- 1
PGs: Prostaglandins
(NSAIDs): Non-steroidal anti-inflammatory drugs
per oral: (p.o.).
