# Supplementary material for: Anti-nociceptive and Anti-inflammatory Activities of Asparacosin A Involve Selective Cyclooxygenase 2 and Inflammatory Cytokines Inhibition: An in-vitro, in-vivo, and in-silico Approach
Source: Front Immunol. 2019 Mar 26;10:581. doi: 10.3389/fimmu.2019.00581 (PMC6443962; doi:10.3389/fimmu.2019.00581)
Supplement: Supplementary file 1 [file Table_1.DOCX]

**HPLC Analysis**

The HPLC method was used for the determination of Aspercosine A in PBS buffer (kept for 24 hours). Chromatographic separation of samples was performed on a C18 column (150mm 4.6 mm; 5 l) using a Perkin Elmer (Norwalk, USA) HPLC system linked with a peristaltic pump series 200, an online degasser, a column oven, a rheodyne 7725 injector, and an UV-Visible detector. Sample (100 µL) were transferred to 2-mL Eppendorf tube and 10 mL was added to each sample. Methanol (200 µL) was added and the tubes were vortexed for 20 sec. The samples were then extracted with mobile phase. The mobile phase was comprised water and methanol (20:80). Flow rate was 1ml/min through a C18 column (10 cm x 4.6 mm) at column oven temperature of 35ºC. The detector wavelength was adjusted at 240 nm. Sample (25 µL) was injected into HPLC system for analysis.


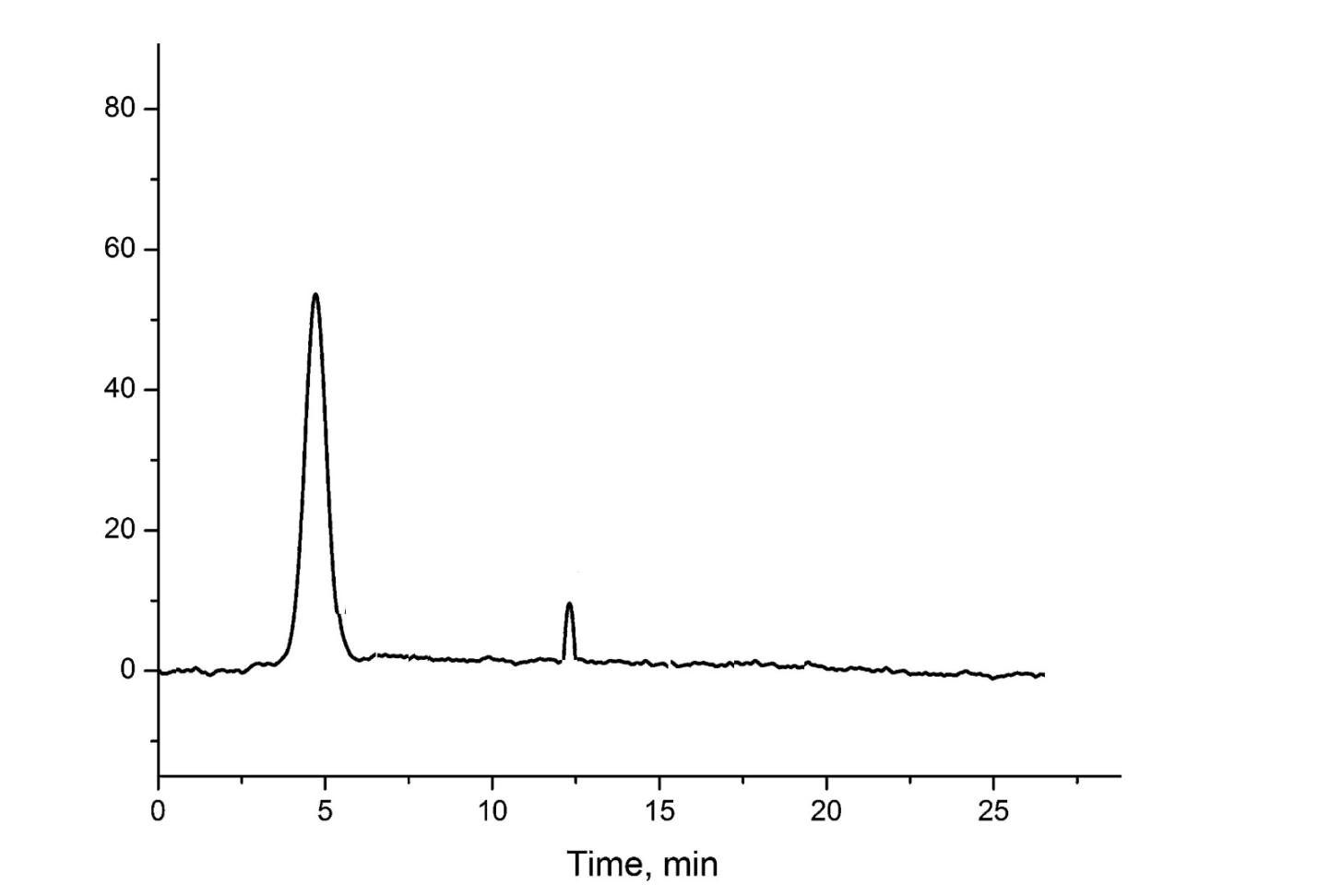


Figure S1: Chromatogram of the Asparacosine A sample after PBS treatment for 24 hours.
